# Risk Factors Associated with Surgical Site Infections: A Retrospective Report from a Developing Country

**DOI:** 10.7759/cureus.4801

**Published:** 2019-06-02

**Authors:** Shahbaz Ansari, Muhammad Hassan, Habiba D Barry, Tariq Ali Bhatti, Syed Zohaib Maroof Hussain, Shah Jabeen, Sundus Fareed

**Affiliations:** 1 Internal Medicine, Jinnah Postgraduate Medical Center, Karachi, PAK; 2 Surgery, Dow University of Health Sciences, Karachi, PAK; 3 Internal Medicine, Dow University of Health Sciences, Karachi, PAK; 4 Surgery, Civil Hospital Karachi, Karachi, PAK; 5 Surgery, Aga Khan University Hospital, Karachi, PAK; 6 Physiology, Liaquat College of Medicine and Dentistry, Karachi, PAK; 7 Internal Medicine, Civil Hospital Karachi, Karachi, PAK

**Keywords:** surgical site infections, clean surgery, risk factors, predictors, predictors of prolonged hospitalization, incidence of ssi, pakistan

## Abstract

Introduction

Any infection occurring at the site of a surgical incision superficially or deep within the fascia, within 30 days of a surgical procedure is termed as a surgical site infection (SSI). Due to limited resources, non-adherence to infection control guidelines and substandard sterilization practices, the incidence is higher in developing countries. The aim of this study is to estimate the incidence of surgical site infections in general surgeries at a tertiary care hospital in Pakistan and identify the predisposing risk factors.

Methods

This was a retrospective analysis that included all surgical records from June 1, 2018, to December 31, 2018. After exclusion, 882 records were included. The incidence of SSI and predisposing risk factors were noted. Data were entered and analyzed using SPSS v. 22.0 (IBM Corp, Armonk, NY, US).

Results

The incidence of SSI was 8.84% (n=78). SSIs were more common in older participants (11.4% vs. 6.4%; p=0.009), in patients with more than 24 hour of preoperative hospital stay (11.2% vs. 64%; p=0.013), in procedures of longer duration (1.53 ± 0.35 vs 2.57 ± 0.17; p<0.0001), and in emergency surgeries (19.2% vs. 7.5%; p=0.0001). The combined incidence of SSIs in American Society of Anesthesiologists (ASA) index III and above was 37 (47.4%) and that in I and II was 41 (52.6%) (p<0.00001).

Conclusion

This study has revealed a very high incidence of surgical site infections. These infections are more common in elderly patients, patients undergoing emergency surgeries, those with longer preoperative hospital stay and longer surgical duration, and patients with a high ASA index.

## Introduction

Any infection occurring at the site of a surgical incision superficially or deep within the fascia, within 30 days of a surgical procedure is termed as a surgical site infection (SSI). If an SSI involves only the skin and underlying subcutaneous tissue, it is termed as a superficial SSI and if it penetrates into the deep fascia and the muscle, it is called a deep SSI [1].

Important patient-related factors increasing the risk of an SSI include pre-existing infection, malnutrition, obesity, low serum albumin, elderly, smoking, and immunosuppression (diabetes mellitus, irradiation). Surgery-related factors include contaminated surgeries, emergency surgeries, prolonged procedures, substandard sterilization, inadequate handling of instruments, and inadequate antiseptic surgical site preparation. Physiological conditions that predispose to an increased incidence of SSI include multi-trauma, hemodynamic instability, shock, massive blood transfusions during the procedure, and postoperative hypothermia, hypoxia, and hyperglycemia. Other independent predictors of SSI include abdominal surgeries, contaminated or dirty procedures, and three or more diagnoses upon hospital discharge [2]. Delayed recognition and inappropriate management of SSI increases the length of hospital stay and treatment cost [3-4]. Patients with SSI have increased the risk of mortality when compared to those without SSI [4].

In a large-scale study conducted by National Healthcare Safety Network (NHSN), which included 850,000 general surgeries from all across United States (US), the incidence of surgical site infection was only 1.9% [5]. On the other hand, in a study conducted in Peshawar, Pakistan, the overall incidence of SSI was 9.29%; 4.88% in clean procedures, 8.39% in clean-contaminated procedures, and 20.45% in contaminated or dirty procedures. The incidence of SSI was significantly higher among patients with American Society of Anesthesiologists (ASA) scores II and III than those with ASA score I in clean-contaminated and dirty procedures [6]. The difference in the incidences of SSI in the two countries is drastic. As mentioned above, SSI is multifactorial and no one factor can be isolated as the causative agent. The higher incidence of SSI in a developing country is not at all surprising. Owing to the limited resources, non-adherence to infection control guidelines, and substandard sterilization practices, the numbers are continuing to rise. This study aimed to estimate the incidence of surgical site infections in general surgeries at a tertiary care hospital in Pakistan and identify the risk factors.

## Materials and methods

This was a retrospective study performed in the surgical department of a tertiary care hospital in Pakistan. All surgical records from June 1, 2018, to December 31, 2018, were extracted and analyzed. The study was approved by the institutional review board.

The surgical records included in the study were of patients of age ≥18 years and performed in accordance with the National Healthcare Safety Network (NHSN). An NHSN procedure is defined as that performed in an operating room where the surgeon makes at least one incision that is closed before leaving the operating room [7]. During the study period, there were 1,129 surgical procedures performed, out of which 1001 fell in the inclusion criteria. Another 119 constituted incomplete and inconsistent data and were eliminated. Hence, 882 surgical records were finally included in this analysis. The patient age, gender, length of preoperative hospital stay, duration of surgery, wound classification, type of surgery (elective vs. emergency), and ASA physical status classification score were recorded (Table 1).

**Table 1:** American Society of Anesthesiologists Physical Status Classification Abbreviation: ASA, American Society of Anesthesiologists; BMI, body mass index; COPD, chronic obstructive pulmonary disease; CHF, congestive heart failure;

ASA 1	A normal healthy patient. Example: Fit, non-obese (BMI under 30), a non-smoking patient with good exercise tolerance
ASA 2	A patient with a mild systemic disease. Example: Patient with no functional limitations and a well-controlled disease (e.g., treated hypertension, obesity with BMI under 35, frequent social drinker, or a cigarette smoker).
ASA 3	A patient with a severe systemic disease that is not life-threatening. Example: Patient with some functional limitation as a result of disease (e.g., poorly treated hypertension or diabetes, morbid obesity, chronic renal failure, a bronchospastic disease with intermittent exacerbation, stable angina, and implanted pacemaker).
ASA 4	A patient with a severe systemic disease that is a constant threat to life. Example: Patient with functional limitation from severe, life-threatening disease (e.g., unstable angina, poorly controlled COPD, symptomatic CHF, recent (less than three months ago) myocardial infarction or stroke.
ASA 5	Moribund patient who is not expected to survive without the operation. The patient is not expected to survive beyond the next 24 hours without surgery. Examples: Ruptured abdominal aortic aneurysm, massive trauma, and extensive intracranial hemorrhage with mass effect.
ASA 6	A brain-dead patient whose organs are being removed with the intention of transplanting them into another patient.

The presence or absence of surgical site infection was recorded. Wounds were classified as clean, clean-contaminated, contaminated, or dirty, as defined by the American College of Surgeons [7]. Data were processed and analyzed through SPSS version 22.0 (IBM Corp, Armonk, NY, USA). Frequencies and percentages were calculated for categorical variables. Mean and standard deviation (SD) were calculated for continuous variables. The overall incidence of SSI was calculated and correlated with predisposing risk factors. Chi-square was applied for correlation; p-value ≤0.05 was taken as significant.

## Results

Of the 882 surgical procedures, 492 (55.8%) were performed on men and 390 (44.2%) on women. The mean age of the participants was 58.2 ± 15.9 years (range: 18-81 years). The mean duration of surgeries was 1.67 ± 1.40 hours. There were 78 (8.84%) cases of SSI identified in this study. SSI was more common in female gender than in male (9.3% vs. 8.5%). Surgical site infections were the most common in contaminated wounds (12.4%) followed by dirty (12.3%), clean-contaminated (10.9%), and clean wounds (7.2%). The results were not statistically significant. SSIs were more common in older participants, in patients with more than 24 hours of preoperative hospital stay, in procedures of longer duration, and in emergency surgeries (p≤0.05). The combined incidence of SSIs in ASA index III and above was 37 (47.4%) and that in I and II was 41 (52.6%) (p<0.00001). All characteristics are shown in Table 2.

**Table 2:** Risk Factors of Surgical Site Infections (N=882)

Variable	Total Participants	Surgical Site Infection		P Value
		No n (%)	Yes n (%)	
Gender				
Male	492 (55.8%)	450 (91.5%)	42 (8.5%)	0.71
Female	390 (44.2%)	354 (90.7%)	36 (9.3%)	
Age (in years)				
≤ 50	451 (51.1%)	422 (93.6%)	29 (6.4%)	0.009
> 50	431 (48.8%)	382 (88.6%)	49 (11.4%)	
Length of Preoperative Hospital Stay				
≤ 24 hour	435 (49.3%)	407 (93.6%)	28 (6.4%)	0.013
> 24 hour	447 (50.7%)	397 (88.8%)	50 (11.2%)	
Mean Duration of Surgery (in hours)				
	882 (100%)	1.53 ± 0.35	2.57 ± 0.17	<0.0001
American Society of Anesthesiologists Physical Status Score				
I	271 (30.7%)	254 (93.1%)	17 (6.9%)	<0.00001
II	437 (49.5%)	413 (91.7%)	24 (8.3%)	
III	128 (14.5%)	102 (86.4%)	26 (13.6%)	
IV-VI	46 (5.2%)	35 (85.4%)	11 (14.6%)	
Wound Classification				
Clean	501 (56.8%)	465 (92.8%)	36 (7.2%)	0.20
Clean-Contaminated	258 (29.3%)	231 (89.1%)	27 (10.9%)	
Contaminated	88 (9.9%)	78 (88.6%)	10 (12.4%)	
Dirty	35 (3.9%)	30 (85.7%)	5 (12.3%)	
Type of Procedure				
Emergency	99 (11.2%)	80 (80.8%)	19 (19.2%)	0.0001
Elective	783 (88.8%)	724 (92.5%)	59 (7.5%)	

## Discussion

This study has reported a relatively higher incidence of SSIs in Pakistan as compared to that of developed countries such as USA (1.9%), France (1.0%), and Italy (2.6%) [5,8-9]. In other developing countries, such as Brazil, the incidence has been reported to be as high as 10% [10]. A local study conducted in another city of Pakistan has reported the incidence of SSI as 9.3% [6].

Our study showed that patients of age above 50 years, with a longer duration of surgery, those who underwent emergency surgeries, and those with a high ASA index were more prone to the development of surgical site infections. The results are comparable to a study conducted in a Brazilian hospital with 16,882 participants. They reported following risk factors for SSI: length of preoperative hospital stay >24 hours; duration of surgery in hours; wound class clean-contaminated, contaminated, and dirty or infected; and ASA index classified as ASA II, III, and IV/V [11]. As with the elderly population, surgical site infections are known to complicate procedures in the older population and increase mortality and the cost of healthcare [12-13]. In another recent local study, the results have been shocking with an SSI incidence as high as 33.6%. Their risk factors included old age, obesity, elective surgeries, the coexistence of diabetes and anemia, and surgeries performed by trainee residents. Perhaps their small sample (n=95) has caused such a drastically high incidence of SSI [14].

In this study, participants who were admitted for more than 24 hours prior to surgery had a greater incidence of SSI. This was also seen in various other studies conducted in both developed and developing countries such as Brazil, Italy, and France [8-9,11]. This association can be explained by the fact that a longer duration of exposure to the healthcare facility prolongs exposure to contamination.

In this study, long-durational surgeries were associated with a higher incidence of SSI. In a study conducted in Brazil in 2017, for each hour of the duration of surgery, the risk of SSI increased by 34% [11]. Similar findings have been seen in studies conducted in developed countries such as Italy and France [8-9]. In a study in the US, a direct correlation between the duration of surgery and 30 days' perioperative complications was seen [15]. This association has been explained in detail by Cheng et al. who have proposed the following reasons: prolonged operative duration renders the incision to be exposed to the environment for a longer time. A prolonged procedure duration also predisposes incisions to tissue desiccation. As the surgery progresses, the tissue concentration of prophylactic antibiotics also decrease and may eventually become inadequate unless the dose is repeated. All these factors increase the risk of surgical site infection [16]. The increased duration of surgery is also associated with many other post-surgical complications such as wound dehiscence, infection in the urinary tract, and even septic shock [15]. This association can be explained by a longer, greater exposure of the bodily structures to open air, to infectious pathogens, or a higher probability of a breach in aseptic technique due to the perforation of the surgeon's gloves in long surgery [17-18].

In this study, SSIs were more in patients with a higher ASA index. The ASA index has been recognized as a risk factor of SSI in many studies [8-9,11]. This can be attributed to poorly controlled factors and the patient in a debilitated state, which makes the risk of SSI higher.

To our knowledge, this study is among the largest ones analyzing surgical site infections in Pakistan. However, because of its retrospective nature, we are unable to establish correlations. Furthermore, it did not include the clinical and microbiological characteristics of the patients and surgical factors, including the type and duration of surgery and the extent of incision. Based on this study, efforts should be made to recognize and eradicate the risk factors associated with a higher incidence of SSI. Elaborated, prospective, randomized trials must be conducted, including clinical, microbiological, and surgical parameters, to establish accurate correlation.

## Conclusions

This study has revealed a very high incidence of surgical site infections. These infections are more common in elderly patients, patients undergoing emergency surgeries, those with a longer preoperative hospital stay and longer surgical duration, and patients with a high ASA Index. Compared to data from the developed world, we have reported a higher incidence of SSIs. Infection control guidelines should be strictly followed and extra measures should be taken in high-risk patients to prevent infections. Long-durational cohorts should be conducted to study the risk factors, pathogens, complications, and outcomes of surgical site infections in depth.
